# HLA-DOB: A Key “Coordinator” Between Cutaneous Melanoma and Psoriasis

**DOI:** 10.7150/jca.110306

**Published:** 2025-07-24

**Authors:** Yingxi Li, Jing Luo, Dongchen Tian, Chenxi Li, Chen Wu, Guixin Wang, Ruitan Guo, Long He, Lin Li, Yao Tian, Lizhi Hu

**Affiliations:** 1 Health Science Center, Ningbo University, Ningbo, Zhejiang, 315211, China.; 2 Immunology Department, Key Laboratory of Immune Microenvironment and Disease (Ministry of Education), Tianjin Medical University, Tianjin, 300070, China.; 3 Tianjin Medical University Cancer Institute and Hospital, National Clinical Research Center for Cancer, Tianjin, 300060, China.; 4 Department of Thoracic Surgery, The Affiliated LiHuiLi Hospital of Ningbo University, Ningbo, Zhejiang, 315040, China.; 5 Department of Dermatology, Tianjin Academy of Traditional Chinese Medicine Affiliated Hospital, Tianjin, 300120, China.

**Keywords:** Cutaneous melanoma, Psoriasis, Mendelian randomization, eQTL, Single-cell transcriptome

## Abstract

**Background:** Psoriasis is a chronic inflammatory skin disease associated with immune dysfunction, and its relationship to cutaneous melanoma is unclear. This study used Mendelian randomization (MR) to explore the causal link between the two and identify risk genes.

**Methods:** SNPs from a psoriasis GWAS (5,072 cases, 478,102 controls) were used as instrumental variables, and melanoma GWAS data (3,751 cases, 372,016 controls) served as the outcome. Causal relationships were assessed using IVW, MR-Egger, and weighted median methods, with sensitivity tests. Co-localization and transcriptome analyses identified risk genes.

**Results:** Forward MR showed psoriasis significantly reduced melanoma risk (PIVW=0.040). The co-localization analysis revealed genes positively associated with the risk of psoriasis, including HLA-DOB, NOTCH4, and VARS2. HLA-DOB was the only risk gene of psoriasis that showed differential expression in cutaneous melanoma based on transcriptional analysis. HLA-DOB was downregulated in melanoma and associated with better prognosis (P=0.033). Single-cell analysis showed that HLA-DOB was mainly enriched in B cells (especially memory B cells) and myeloid cells (particularly DC: monocyte-derived).

**Conclusion:** Our findings suggest an inverse causal relationship between melanoma and psoriasis. Importantly, we also found that HLA-DOB can be served as a key “coordinator” between cutaneous melanoma and psoriasis: a risk gene of psoriasis and a protective factor of cutaneous melanoma.

## Introduction

Psoriasis (PsO) is a common, chronic and relapsing immune-related inflammatory dermal disease characterized by red plaques and silvery scales on the skin surface 1. Approximately 503.62 people per 100,000 population suffer from psoriasis worldwide 2. Currently, there are various regimens for moderate to severe psoriasis. However, some treatment options still have limitations. Conventional phototherapy, Methotrexate (MTX), Cyclosporine (CsA) and biological agents (tumor necrosis factor α inhibitors) may increase the risk of tumors 3. Cutaneous melanoma (SKCM) is a malignant skin tumor originating from melanocytes. Its basic cause is usually DNA damage, and the inducing factors include excessive ultraviolet exposure, multiple moles, fair skin, history of sunburn and poor immune system 4-6. Among them, an important external factor is the exposure to ultraviolet radiation, especially intermittent sunlight exposure 7.

Currently, the relationship between PsO and SKCM is still controversial. A retrospective study of 72,739 patients showed that the incidence of melanoma among those with psoriasis was 1.8% (95% confidence interval: 1.5-2.2%), while the incidence of melanoma in patients without skin diseases was 4.5% (95% confidence interval: 3.8-5.4%), suggesting that the risk of melanoma in patients with psoriasis is 40% lower than that in patients without skin diseases 8. However, a cohort study of 7,061 psoriasis patients showed that the risk of melanoma in patients with psoriasis was about 3.75 times higher than that in the general population. The risk was notably greater in those with severe psoriasis, showing a standardized incidence rate ratio of 11.01 9. A retrospective cohort study by Reddy *et al.* also found that patients with psoriasis had a 53% increased risk of melanoma compared with non-psoriatic patients. The increased risk of malignant tumors does not affect the prognosis of psoriasis patients 10. In addition, a systematic review by Pouplard *et al.* also found no increased risk of melanoma in psoriasis patients 11. Therefore, it is necessary to clarify the relationship between PsO and SKCM with a novel method.

Mendelian randomization (MR) is an epidemiological method of instrumental variable analysis to assess the causal relationship between modifiable exposures or risk factors and clinically relevant outcomes 12. The principle of MR is based on Mendel's second law that genetic alleles are independently separated when DNA is passed from parents to offspring during gamete formation 13. The alleles of this exposure-related genetic variation are randomly assigned, reducing the impact of environmental confounding and reverse causality. In view of this, single nucleotide polymorphisms (SNPs) are often used as instrumental variables for putative risk factors 14. Compared with traditional randomized controlled trials, MR has many advantages, such as faster and cheaper, revealing potential causal relationships, avoiding ethical issues, and reflecting lifelong perturbation effects 13.

In this study, we used bidirectional two-sample MR to reveal the causal relationship between PsO and SKCM. MR results showed that the elevated PsO reduced the risk of SKCM. However, there was no significant causal relationship of SKCM on the risk of PsO. Further, eQTL co-localization analysis, transcriptome analysis, and single-cell analysis were conducted to deeply explore the relationship. Notably, we found that HLA-DOB is a risk factor of PsO from co-localization analysis. While, HLA-DOB is expressed at a low level in SKCM, and the upregulation of HLA-DOB could improve the overall survival of patients with SKCM in transcriptome analysis. From the single-cell analysis, we further found that HLA-DOB exhibits immune cell-specific expression in SKCM, mainly enriched in B cells (especially memory B cells) and myeloid cells (particularly DC: monocyte-derived). In summary, our study found a causal relationship between PsO and SKCM, in which HLA-DOB played an important role.

## Methods

### 1. Bidirectional MR analysis

#### 1.1. MR study design

We constructed bidirectional MR analyses to elucidate the causal relationship between PsO and SKCM. Forward MR used PsO as the exposure and SKCM as the outcome. Conversely, reverse MR used SKCM as the exposure and PsO as the outcome. All included instrumental variables followed three MR assumptions: (1) there is a strong correlation between genetic variants and exposure factors; (2) there is little association between genetic variants and potential confounders; and (3) genetic variants cannot directly affect outcome factors.

#### 1.2. GWAS data sources

The GWAS summary statistics data for psoriasis vulgaris employed in this investigation originated from the GWAS Catalog (ebi-a-GCST90018907) 15. In our study, the identified PsO phenotype encompassed 24,211,145 single nucleotide polymorphisms (SNPs) derived from 5,072 patients and 478,102 control individuals of European ancestry (Finland, U.K.), as well as 206 patients and 172,289 controls of East Asian ancestry. The GWAS summary statistics data for SKCM were acquired from the UK Biobank through the IEU Open GWAS project (IEU GWAS ID: ieu-b-4969). The identified phenotype for SKCM comprised 11,396,019 SNPs derived from 3,751 patients and 372,016 control individuals of European ancestry.

#### 1.3. SNPs selection

Referring to studies 16-18, we determined SNPs that reached the genome-wide significance threshold (P < 1.0 x 10-8) as instrumental variables (IV) to determine the relationship between PsO and SKCM. SNPs are selectively retained based on their clustering within genomic regions that are 10,000 kilobase pairs (kbp) apart and in linkage disequilibrium (R^2^ < 0.001), ensuring the independence of selected SNPs. Subsequently, we calculated the F statistic of the selected SNPs and selected SNPs with F > 10 as strong instrumental variables. When performing forward MR analysis with PsO as exposure and SKCM as outcome, we identified 11 SNPs. In contrast, when performing reverse MR analysis with SKCM as exposure and PsO as outcome, we identified 10 SNPs.

#### 1.4. Statistical analysis

In our study, we evaluated the bidirectional causal relationship between PsO and SKCM using the R package “TwoSampleMR” with different MR methods, including inverse variance weighted (IVW), MR-Egger, weighted median, and simple weighted median (VM). Among them, IVW was selected as the main research method because it was consistent across all effective genetic variants and had the greatest testing power 19. The efficiency of weighted median is the same as IVW, but it should be noted that the weighted median method has certain limitations, and the robustness of weighted median can only be maintained when the effective genetic variants account for half of the total weight 20. The efficiency of the MR-Egger method is significantly lower than that of the IVW and weighted median methods 20. Since MR-Egger relies on the InSIDE (Instrument Strength Independent of Direct Effects) assumption, this assumption has inherent limitations in terms of satisfaction. The validity of MR-Egger may be challenged when all genetic variants are null or the distribution of pleiotropic effects is not independent of instrument strength 20. Subsequently, we performed sensitivity analyses to assess the robustness of the Mendelian randomization (MR) results. Cochrane's Q value was used to assess heterogeneity, and funnel plots were used to visualize heterogeneity. MR-PRESSO analysis was used to analyze horizontal pleiotropy with 'MR-PRESSO' R packages. MR Egger intercept analysis was used to assess direct pleiotropy. This method uses the deviation of the intercept from zero in MR Egger regression as the effect size of direct pleiotropy 21. To further assess the specific effect of each SNP, we performed a leave-one-out analysis, which systematically excluded individual SNPs from the analysis to estimate their respective causal effects.

### 2. Co-localization analysis

We employed colocalization analysis to uncover risk genes for PsO. Expression quantitative trait loci (eQTL) describe the genetic link between SNPs and gene expression levels 22. GWAS describe the association between SNPs and specific traits or diseases 23. If a molecular trait is causally related to a complex phenotype, then any genetic variation that affects the molecular trait will also affect the phenotype. Based on this assumption, by combining eQTL data from different tissue types with patterns of association in GWAS, colocalization analysis revealed potential genes shared between GWAS signals and eQTLs. Compared to previous eQTL methodologies applied in GWAS analysis, Sherlock approach utilizes gene expression SNPs in both cis- and trans-regulatory regions, discerns causality from coincidence, and exhibits generalizability across various molecular traits 24. Based on the Logarithmic Bayes Factor (LBF) value, we pinpointed target genes within risk loci for PsO. The higher the LBF, the stronger the association between the locus and the gene. For colocalization analysis, we utilized GWAS data of PsO (“ebi-a-GCST90018907”) and eQTL data of two skin types (“GTEx V7 Sun-unexposed (suprapubis)” and “GTEx V7 Sun-exposed (calf)”) from the Genotype-Tissue Expression (GTEx) database 15, 25. Colocalization analysis was constructed using the “coloc” R package and the web tool Sherlock.

### 3. Transcriptomic analysis

Based on transcriptome profiling data obtained from TCGA and downloaded via “BiocManager” R package, we conducted an analysis to investigate the correlations between the expression of PsO risk genes and molecular as well as clinical features in SKCM 26. Initially, we identified the differentially expressed genes in SKCM using a significance threshold of p < 0.05 and a fold change of 2, employing the “limma” R package for the analysis of differential expression between SKCM and normal samples. We then crossed these genes with PsO risk genes obtained by colocalization analysis. Finally, PsO risk genes (HLA-DOB) showing significant differential expression in SKCM were identified. This result was visualized using Venn diagrams created with the “ggVennDiagram” and “ggplot2” R packages. Then, we used the surv_cutpoint() function from the “survminer” R package to determine the optimal cutoff value for HLA-DOB expression. Based on this value, SKCM patients were classified into high and low expression groups. Furthermore, the relationship between HLA-DOB expression levels and overall survival (OS) time in SKCM patients was analyzed using Kaplan-Meier, which was implemented with the “survival” R package. We used Genemania to identify the co-expressed genes of HLA-DOB 27, and used STRING to construct the protein-protein interaction of HLA-DOB (PPI) network 28. Furthermore, we performed gene ontology (GO) enrichment analysis using the “clusterProfiler” R package to evaluate the potential biological overcompetence of HLA-DOB 29-31. Visualization of the above results was achieved using the “ggplot2” R package.

### 4. Single-cell transcriptome analysis

We collected 10X single-cell sequencing profiles on specimens sourced from GSE215120 dataset 32, 33. Low-quality single cells were removed through the following criteria: 1. nCount_RNA > 500 & nFeature_RNA >300. 2. the proportion of mitochondrial genes counts (< 15%) and hemoglobin gene counts (< 1%). A total of 24362 cells were retained for subsequent analysis, with an average detection of 2162 genes/cell. We used the "Seurat" R package for dimensionality reduction and unsupervised clustering, and used the "harmony" package to eliminate batch effects between multiple samples. Subsequently, UAMP algorithms was used to obtain cascade maps, revealing 15 major cell clusters. Cell types were further identified through manual annotation using the CellMarker database 34. To investigate HLA-DOB expression across cell types, we utilized the FindAllMarkers function in the Seurat package. Finally, we conducted a subpopulation analysis focusing on B cells and myeloid cells. UMAP was reapplied to these subsets, and cell types were annotated using the CellMarker database. The expression levels of HLA-DOB in these subpopulations were further analyzed using the FindAllMarkers function in Seurat.

### 5. Immunohistochemistry (IHC)

Tissues were deparafnized, rehydrated, and permeated using Triton-X100 (T8200, Solarbio, Beijing, China) and followed by antigen retrieval using EDTA Antigen Retrieval solution (c1034, Solarbio, Beijing, China). The sections were incubated with Anti-HLA-DOB antibody (sc-69739, Santa-cruz, USA) at 4 °C overnight followed by a biotinylated secondary antibody (diluted at 1:200) at RT for 60 min. Then, the sections were stained with DAB staining solution (AR1022, BOSTER Biological Technology, Wuhan, China).

### 6. Statistical analysis

All statistical analyses were performed using R software (v4.2.3). P values < 0.05 were considered statistically significant.

## Results

### An inverse causal effect of PsO on the risk of SKCM in forward MR analysis

**Figure 1:**
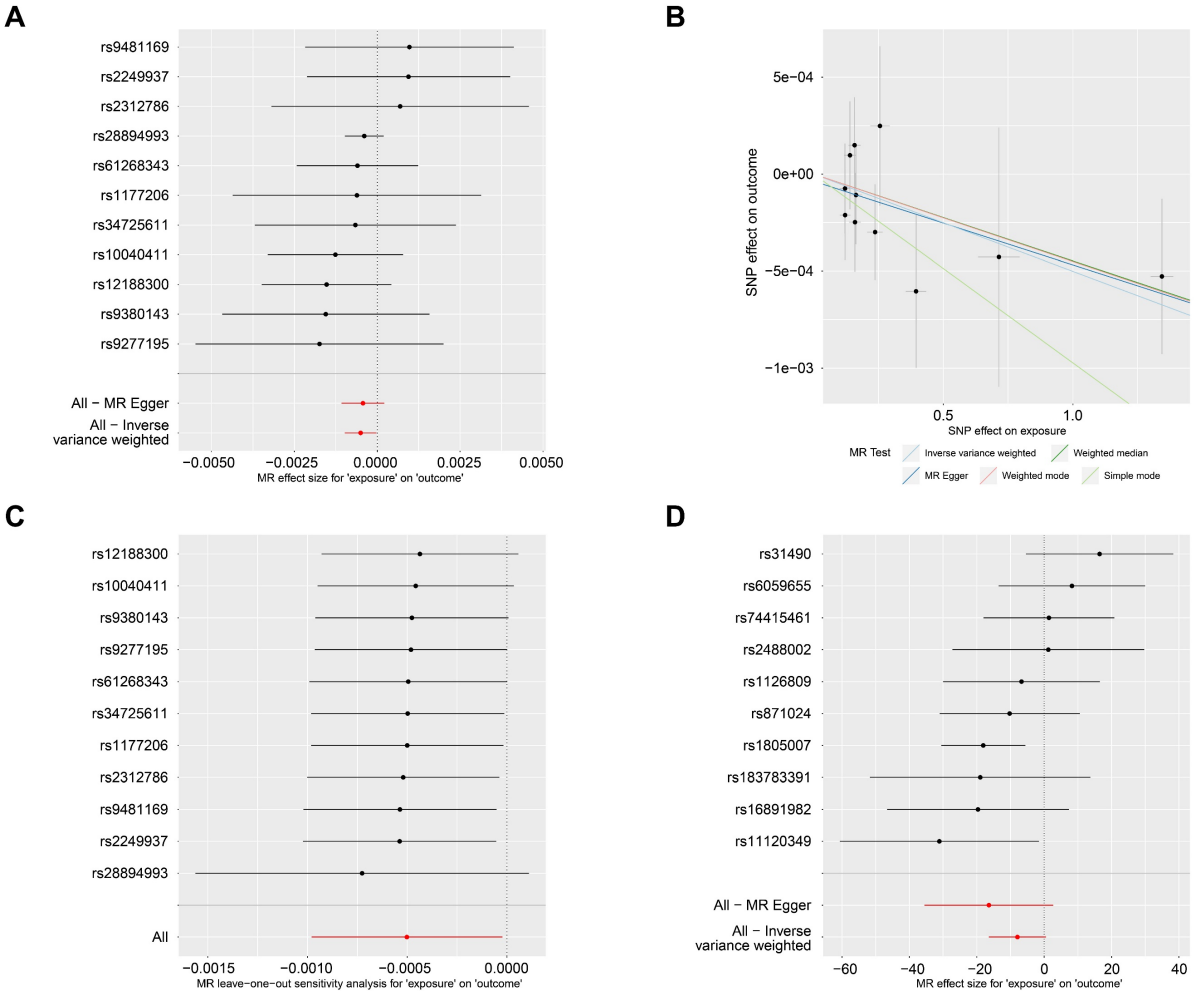
** The causal relationship of PsO and SKCM by MR analysis. (A)** Forest plot displaying the effect estimates of genetic variants associated with PsO on SKCM risk. Each line represents a SNP, with the red line indicating the overall effect estimate. **(B)** Scatter plot showing the relationship between the genetic associations with PsO and SKCM for each SNP. The regression lines represent different MR methods, including Inverse Variance Weighted, Weighted Median, MR-Egger, Weighted mode and Simple mode. **(C)** Forest plot representing the results of the MR leave-one-out sensitivity analysis for the effect of PsO on SKCM. **(D)** Forest plot displaying the effect estimates of genetic variants associated with SKCM on PsO risk. Each line represents a SNP, with the red line indicating the overall effect estimate.

In the forward MR analysis, PsO was set as the exposure factor, and SKCM as the outcome factor. There were 11 SNPs were found and identified as instrumental variables (Fig. 1A). The forward MR analysis revealed a significant causal effect between PsO and SKCM (P_IVW_= 0.040; table 1). In Fig. 1B, scatterplots showed that the increased risk of PsO was associated with the decreased risk of SKCM with five MR methods. Furthermore, we found there was no heterogeneity according to Cochran's Q analysis (Cochran's Q_MR-Egger_ = 4.565 and P = 0.870, Q _IVW_ = 4.665 and P = 0.912; Table 1). And there was no pleiotropy affecting the association according to the direct pleiotropy analysis (P_MR-Egger-intercept_= 0.758; Table 1). Similar results were also verified by horizontal pleiotropy tests (P_MR-PRESSO_= 0.895; Table 1). In Fig. 1C, the leave-one-out test showed that the extreme value of any SNPs did not affect the overall positive MR results.

### No causal effect of SKCM on the risk of PsO in reverse MR analysis

When it comes to the reverse MR analysis, SKCM was set as the exposure factor, and PsO as the outcome factor. 10 SNPs were identified as instrumental variables (Fig. 1D). However, the reverse MR analysis showed that there is no causal effect of SKCM on PsO. (P_IVW_ = 0.067; Table 2).

### Risk loci of PsO with co-localization analysis

In order to find target genes and risk loci related to PsO, we utilized skin-related expression quantitative trait loci (eQTL) data for a co-localization investigation. eQTL data are tissue-specific and can identify regions in the genome that harboring sequence variants and influencing gene expression 35. Therefore, in our analysis, we combined the eQTL data of “GTEx V7 Sun Exposed (Crucula)” with the PsO GWAS data “ebi-a-GCST90018907”. And the analysis revealed several genes that were significantly positively associated with the expression of PsO risk loci in sun-exposed skin (P < 0.05, LBF > 0; Table S1), including HLA-DOB (P < 0.001, LBF = 7.60), NOTCH4 (P < 0.001, LBF = 14.90) and VARS2 (P < 0.001, LBF = 15.09) (Table S1). Meanwhile, we combined the eQTL data of “GTEx V7 Not Sun Exposed (Suprapubic)” with the PsO GWAS data “ebi-a-GCST90018907”. This analysis revealed several genes that were significantly positively associated with the expression of PsO risk loci in not sun-exposed skin (P < 0.05, LBF > 0; Table S2), including HLA-DOB (P < 0.001, LBF = 7.60), HLA-DQB1 (P < 0.001, LBF = 15.20), and CYP21A2 (P < 0.001, LBF = 15.08) (Table S2).

### HLA-DOB, a risk locus of PsO and a protective factor of SKCM

**Figure 2:**
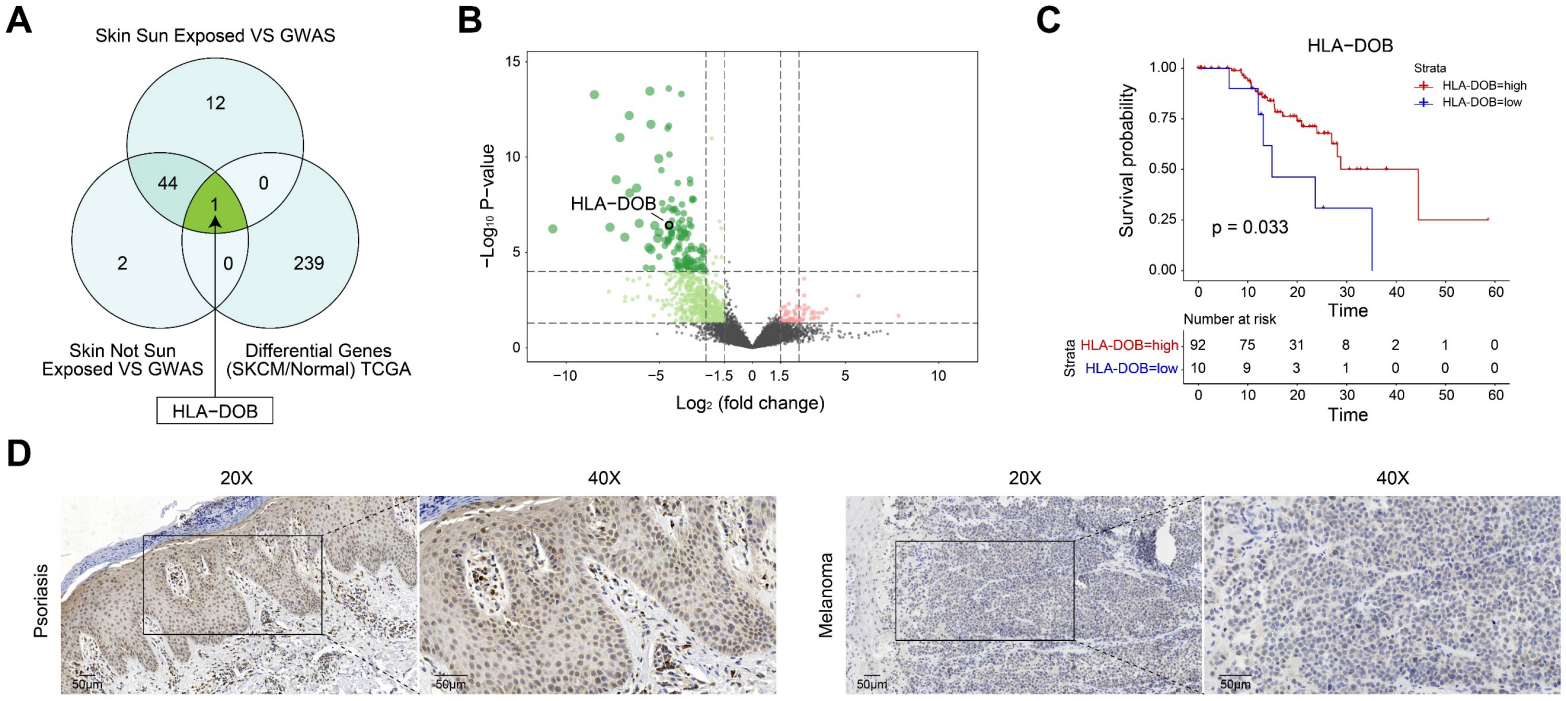
** Target genes between PsO and SKCM. (A)** Venn diagram showing the overlap of all three groups. HLA-DOB is highlighted as a key gene in the intersection.'Skin Sun Exposed VS GWAS' presents genes identified from the conjunction analysis between eQTL data from 'GTEx V7 Sun Exposed (Lower leg)' and PsO GWAS data 'ebi-a-GCST90018907'.'Skin Not Sun Exposed VS GWAS' presents genes identified from the conjunction analysis between eQTL data from 'GTEx V7 Not Sun Exposed (Suprapubic)' and PsO GWAS data 'ebi-a-GCST90018907'.'Differential Genes (SKCM/Normal) TCGA'presents genes DEGs between SKCM and normal tissue form TCGA database. **(B)** Volcano plot illustrating differentially expressed genes between SKCM and normal tissue form TCGA database. **(C)** Kaplan-Meier survival curve comparing overall survival rates between high and low HLA-DOB expression groups in SKCM patients. **(D)** The expression of HLA-DOB by immunohistochemistry analysis in psoriasis patients and SKCM patients.

To further elucidate the relationship between PsO risk genes in two types of normal skin tissues and SKCM, we identified differential gene expression between SKCM and normal samples using TCGA data (Table S3). Subsequently, we intersected these genes with PsO risk genes obtained from two distinct types of skin tissues. Specifically, only HLA-DOB exhibited differential expression in SKCM among non-sun-exposed skin tissues. While in sun-exposed skin tissues, HLA-DOB was the only risk gene of PsO showing differential expression in SKCM (Fig. 2A). As shown by the volcano plot, HLA-DOB expression was significantly downregulated in SKCM compared with normal samples (Fig. 2B). Meanwhile, the Kaplan-Meier survival analysis for SKCM was conducted, which demonstrated a positive correlation between upregulated expression levels of HLA-DOB with favorable overall survival in SKCM patients (P = 0.033) (Fig. 2C). To verify it in clinical samples of patients, we performed immunohistochemistry and found that HLA-DOB is higher expressed in psoriatic skin tissues, while expressed low in SKCM (Figure 2D). Therefore, HLA-DOB acted as a protective factor of SKCM. However, as we mentioned above, HLA-DOB is a risk loci of PsO.

### Molecular mechanisms and functional annotations of HLA-DOB

**Figure 3:**
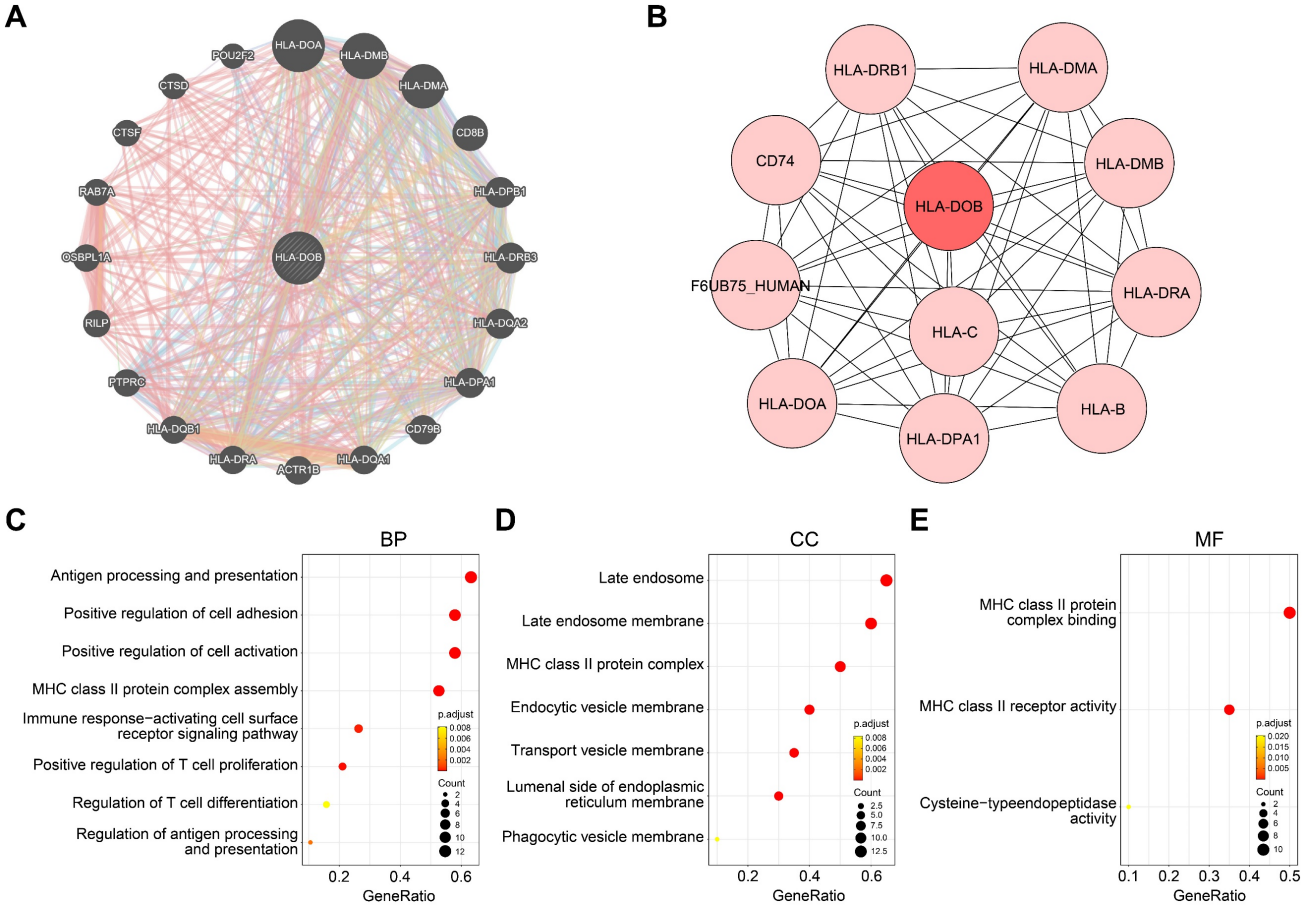
** Molecular mechanisms and functional annotations of HLA-DOB. (A)** Co-expression network plot revealed interactions between HLA-DOB and its neighboring genes. **(B)** PPI network plot highlighting HLA-DOB and its direct connections with protein. **(C-E)** The enrichment levels of HLA-DOB and its neighboring genes in biological processes **(C)**, cellular components **(D)** and molecular functions **(E)**.

To elucidate the molecular mechanisms of HLA-DOB in SKCM, we identified co-expressed genes closely associated with HLA-DOB and constructed a PPI network for HLA-DOB. Co-expression analysis revealed that HLA-DOB is primarily linked to the expression of immune-related genes such as HLA-DOA, HLA-DMB, and HLA-DMA (Fig. 3A). The PPI network further validated the molecular-level correlation of HLA-DOB with these genes (Fig. 3B). To develop the biological functions of HLA-DOB, we conducted GO enrichment analysis to assess the enrichment levels of HLA-DOB and its neighboring genes. Antigen processing and presentation (GO:0019882), regulation of antigen processing and presentation (GO:0002577); MHC class II protein complex assembly (GO:0002399); positive regulation of T cell proliferation (GO:0042102), and regulation of T cell differentiation (GO:0045580) were enriched in biological processes (BP) (Fig. 3C). MHC class II protein complex (GO:0042613); endocytic vesicle membrane (GO:0030666), and transport vesicle membrane (GO:0030658) were enriched in cellular components (CC) (Fig. 3D). MHC class II protein complex binding (GO:0023026) and MHC class II receptor activity (GO:0032395) were enriched in molecular functions (MF) (Fig. 3E). Taken together, the above findings revealed the immunological relevance of HLA-DOB.

### Cell type-specific expression of HLA-DOB in SKCM tissues

**Figure 4:**
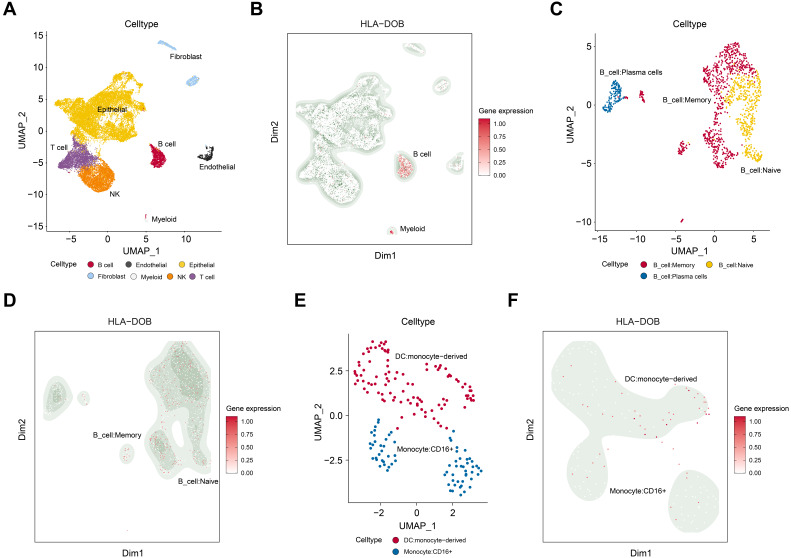
** The immune characterize of HLA-DOB in SKCM with single-cell RNA sequencing analysis. (A)** UMAP plot illustrating the cell type distribution in SKCM, including B cells, endothelial cells, epithelial cells, fibroblasts, myeloid cells, NK cells, and T cells. **(B)** Feature plot of HLA-DOB expression in different cell types of SKCM. The color intensity represents the level of HLA-DOB expression. **(C)** UMAP plot illustrating the cell type distribution of B cell subsets in SKCM, including B_cells:Memory, B_cells:naive, and B_cells:plasma_cells. **(D)** Feature plot of HLA-DOB expression in different types of B cell subsets in SKCM. The color intensity represents the level of HLA-DOB expression. **(E)** UMAP plot illustrating the cell type distribution of myeloid cells subset in SKCM, including DC: monocyte-derived and Monocyte: CD16+. **(F)** Feature plot of HLA-DOB expression in different types of myeloid cell subsets in SKCM. The color intensity represents the level of HLA-DOB expression.

We performed dimensionality reduction and clustering on the GSE215120 dataset and annotated the 15 identified cell clusters using the CellMarker database. UMAP visualization revealed distinct cell populations, including B cells, myeloid cells, endothelial cells, epithelial cells, fibroblasts, and other cell types (Fig. 4A). Notably, we observed a significant increase in HLA-DOB expression in B cells and myeloid cells within SKCM tissues (Fig. 4B).

To further investigate this, we conducted a subpopulation analysis of B cells and myeloid cells. UMAP visualization demonstrated that B cells were subdivided into memory B cells, naive B cells, and plasma cells (Fig. 4C). HLA-DOB predominantly expressed in memory B cells (Fig. 4D). Similarly, myeloid cells were mainly classified into DC: monocyte-derived and Monocyte: CD16^+^ (Fig. 4E). HLA-DOB primarily enriched in DC: monocyte-derived (Fig. 4F).

## Discussion

Currently, in the existing observational studies, the relationship between PsO and SKCM is still controversial. We employed a bidirectional MR analysis approach combined with eQTL and single-cell transcriptomic analysis for the first time to investigate the causal relationship between PsO and SKCM. The choice of MR in this study was driven by its ability to leverage genetic variants as instrumental variables for robust causal inference, effectively minimizing confounding and reverse causation. Unlike observational studies, which can only show associations, MR provides evidence of causality. Compared to randomized controlled trials (RCTs), MR is more cost-effective and ethically feasible, especially when studying harmful exposures. Additionally, while traditional genetic studies like GWAS identify genetic associations, MR builds on these findings to infer causality. Our study analysis found a significant causal effect of the increased risk in PsO on the decreased risk in SKCM. Through co-localization analysis, we identified HLA-DOB upregulation as a risk factor of PsO. Transcriptome analysis showed that HLA-DOB has a potential role in SKCM suppression and was significantly positively correlated with overall survival, which may be related to the anti-tumor immune response. Single-cell analysis revealed cell-specific expression of HLA-DOB in SKCM, enriched in B cells and myeloid cells. In summary, HLA-DOB acted as an important “coordinator” between PsO and SKCM.

When it comes to the HLA-DOB, the role of HLA-DOB in cancers remains uncertain. Chen *et al.* used GEPIA analysis to reveal an association between HLA-II genes (particularly HLA-DOB) and overall survival in clinical melanoma patients. They covered 21 common cancers in the TCGA database, ranked HLA-II genes as the most important gene for overall survival in melanoma patients compared with other types of cancer 36. Wu *et al.* identified that HLA-DOB and HLA-DQB2 genes acted as a tumor suppressor with better prognosis. And Li *et al.* found that HLA-DOB was expressed in the ovarian cancer group with higher immune infiltration and associated with prolonged overall survival 37. Another study also further confirmed through multivariable Cox regression that high levels of HLA-DOB expression can be used as an independent predictor of overall survival in patients with ovarian cancer 38. High HLA-DOB expression reduces the risk of death in ovarian cancer patients by 32%." (HR = 0.68, P = 0.001134) 38. However, Pu *et al.* showed that HLA-DOB is associated with poor prognosis and significantly increased mortality in patients with advanced non-small cell lung cancer receiving first-line chemotherapy 39. Baran *et al.* further showed that HLA-DOB promotes tumor progression by regulating the infiltration of cancer-associated fibroblasts (CAFs) and M2 macrophages 40.

In our study, we confirmed that HLA-DOB is co-expressed with other HLA class II genes and have interaction with them (Figure 3A-B). In addition, functional enrichment analysis confirmed that HLA-DOB is mainly involved in the processing and presentation of antigens such as MHC class II protein complex binding and MHC class II receptor activity (Figure 3C-E). Single-cell analysis confirmed that HLA-DOB is enriched in B cells (especially memory B cells) and myeloid cells (particularly DC: monocyte-derived) (Figure 4B-F). From Denzin's study, they found that HLA-DOB and HLA-DOA bind to HLA-DO molecules, thereby affecting the presentation of MHC class II antigens in B cells by inhibiting the activity of HLA-DM 41. Other studies further showed that high expression of MHC-II antigens is associated with increased metastatic spread, advanced tumor stage and reduced survival 42-44. Therefore, we hypothesized that HLA-DOB inhibits the progression of SKCM by suppressing antigen processing and presentation by MHC class II factors, which is consistent with our finding that HLA-DOB improves the prognosis of SKCM patients.

Our study found a causal relationship between PsO and SKCM and identified a key “coordinator” HLA-DOB for them with potential clinical prediction. Through molecular mechanism and biological function studies, we found that MHC-II antigen processing and presentation may be the downstream pathway for HLA-DOB to exert its effects. Further research is needed on the mechanism of action of MHC-II antigen processing and presentation in the relationship between HLA-DOB, PsO and SKCM.

## Supplementary Material

Supplementary table 1.

Supplementary table 2.

Supplementary table 3.

## Figures and Tables

**Table 1:** Forward MR results of PsO as the exposure and SKCM as the outcome.

MR Results	Method	nsnp	beta	se	pval
	MR Egger	11	-0.0004	0.0003	0.2224
	Weighted median	11	-0.0005	0.0003	0.1080
	Inverse variance weighted	11	-0.0005	0.0002	0.0400
	Simple mode	11	-0.0010	0.0006	0.1628
	Weighted mode	11	-0.0005	0.0003	0.1579
Heterogeneity test	Method	Q	Q_df	Q_pval	
	MR Egger	4.5647	9	0.8705	
	Inverse variance weighted	4.6653	10	0.9124	
Direct pleiotropy	Egger_intercept	se	pval		
	-3.68E-05	0.0001	0.7583		
Horizontal pleiotropy	Pvalue of MR-PRESSO results				
	0.895				

**Table 2:** Reverse MR results of SKCM as the exposure and PsO as the outcome.

MR Results	Method	nsnp	beta	se	pval
	MR Egger	10	-16.4101	9.7561	0.1311
	Weighted median	10	-9.9309	5.2164	0.0569
	Inverse variance weighted	10	-7.9365	4.3263	0.0666
	Simple mode	10	-13.7174	9.6613	0.1894
	Weighted mode	10	-15.0529	6.9737	0.0592
Heterogeneity test	Method	Q	Q_df	Q_pval	
	MR Egger	12.8077	8	0.1186	
	Inverse variance weighted	14.3135	9	0.1116	
Direct pleiotropy	Egger_intercept	se	pval		
	2.55E-02	0.0263	0.3605		
Horizontal pleiotropy	Pvalue of MR-PRESSO results				
	0.113				

## Abbreviations

PsO: Psoriasis
SKCM: Cutaneous Melanoma
CsA: Cyclosporine
MTX: Methotrexate
MR: Mendelian Randomization
SNP: Single Nucleotide Polymorphism
GWAS: Genome-Wide Association Study
eQTL: Expression Quantitative Trait Loci
IV: Instrumental Variables
Kbp: Kilobase Pairs
IVW: Inverse-Variance Weighted
VM: Simple Weighted Median
InSIDE: Instrument Strength Independent of Direct Effects
TCGA: The Cancer Genome Atlas
LBF: Logarithmic Bayes Factor
GTEx: Genotype-Tissue Expression
OS: Overall Survival
PPI: Protein-Protein Interaction
GO: Gene Ontology
t-SNE: t-Distributed Stochastic Neighbor Embedding
UMAP: Uniform Manifold Approximation and Projection
MHC: Major Histocompatibility Complex
HR: Hazard Ratio
HLA: Human Leukocyte Antigen
CAFs: Cancer-Associated Fibroblasts
